# Executive Summary from the 2017 Emergency Medicine Resident Wellness Consensus Summit

**DOI:** 10.5811/westjem.2017.9.36182

**Published:** 2018-02-19

**Authors:** Nicole Battaglioli, Felix Ankel, Christopher I. Doty, Arlene Chung, Michelle Lin

**Affiliations:** * Mayo Clinic, Department of Emergency Medicine, Rochester, Minnesota; † HealthPartners Institute, Department of Emergency Medicine, University of Minnesota Medical School, St Paul, Minnesota; ‡ University of Kentucky, Department of Emergency Medicine, Lexington, Kentucky; § Icahn School of Medicine at Mount Sinai, Department of Emergency Medicine, New York, New York; ¶ University of California San Francisco, Department of Emergency Medicine, San Francisco, California

## Abstract

**Introduction:**

Physician wellness has recently become a popular topic of conversation and publication within the house of medicine and specifically within emergency medicine (EM). Through a joint collaboration involving Academic Life in Emergency Medicine’s (ALiEM) Wellness Think Tank, Essentials of Emergency Medicine (EEM), and the Emergency Medicine Residents’ Association (EMRA), a one-day Resident Wellness Consensus Summit (RWCS) was organized.

**Methods:**

The RWCS was held on May 15, 2017, as a pre-day event prior to the 2017 EEM conference in Las Vegas, Nevada. Seven months before the RWCS event, pre-work began in the ALiEM Wellness Think Tank, which was launched in October 2016. The Wellness Think Tank is a virtual community of practice involving EM residents from the U.S. and Canada, hosted on the Slack digital-messaging platform. A working group was formed for each of the four predetermined themes: wellness curriculum development; educator toolkit resources for specific wellness topics; programmatic innovations; and wellness-targeted technologies.

**Results:**

Pre-work for RWCS included 142 residents from 100 different training programs in the Wellness Think Tank. Participants in the actual RWCS event included 44 EM residents, five EM attendings who participated as facilitators, and three EM attendings who acted as participants. The four working groups ultimately reached a consensus on their specific objectives to improve resident wellness on both the individual and program level.

**Conclusion:**

The Resident Wellness Consensus Summit was a unique and novel consensus meeting, involving residents as the primary stakeholders. The summit demonstrated that it is possible to galvanize a large group of stakeholders in a relatively short time by creating robust trust, communication, and online learning networks to create resources that support resident wellness.

## INTRODUCTION

Physician wellness has recently become a popular topic of conversation and publication within the house of medicine and specifically within emergency medicine (EM).^1^,^2^,^3^,^4^,^5^ Multiple major organizations in EM have tackled the issue of physician wellness and burnout, including the 2017 Emergency Medicine Wellness and Resiliency Summit that convened key leaders from those organizations. The purpose of this summit was to identify areas of overlap and synergy so that collaborative projects and possibly best practices could be established for emergency physician wellness.^6^

National organizations, such as the Accreditation Council on Graduate Medical Education, have recently placed a high priority on resiliency and wellness in trainees.^7^ Similar efforts have been undertaken by the American Medical Association,^8^ the American College of Emergency Physicians,^9^ and the American Academy of Emergency Medicine.^10^ Mirroring recent literature showing that emergency physicians are at particularly high risk of burnout syndrome,^11^ the rate of burnout among trainees is as high as 60%.^12^

Several recent studies have identified factors associated with increased resiliency^13^,^14^ with one meta-analysis demonstrating several interventions associated with increased resiliency and lower incidence of burnout syndrome in graduate medical education.^15^ No literature, however, has focused exclusively on the high-risk burnout population of EM residents. Through a joint collaboration involving Academic Life in Emergency Medicine’s (ALiEM) Wellness Think Tank, Essentials of Emergency Medicine (EEM), and the Emergency Medicine Residents’ Association (EMRA), a one-day Resident Wellness Consensus Summit (RWCS) was organized. This summit primarily convened a group of essential stakeholders to the conversation, EM residents, to clarify the present state of wellness initiatives among EM training programs, and potentially identify best practices and tangible tools to increase physician wellness. To our knowledge, this is the first national consensus event of its kind comprised primarily of residents focusing on resident wellness.

## METHODS

### Pre-Conference Preparation

Seven months before the RWCS event, pre-work began in the ALiEM Wellness Think Tank, which was launched in October 2016. The Wellness Think Tank is a virtual community of practice involving EM residents from 100 different training programs in the U.S. and Canada, hosted on the Slack digital-messaging platform (Slack Technologies). A core mission of the community was to convene EM residents in a way that allowed for cross-institutional communication and collaboration. A working group was formed for each of the four themes: wellness curriculum development; lesson plans for specific wellness topics; programmatic innovations; and wellness-targeted technologies. Ideas, conversations, and materials were shared with the broader Wellness Think Tank community ahead of the conference to inform consensus opinions.

### RWCS Event

The RWCS was held on May 15, 2017, as a pre-day event prior to the 2017 EEM conference in Las Vegas, Nevada. The event was promoted on EEM’s home page, ALiEM blog, the Council of Emergency Medicine Residency Directors and EMRA email listservs, Twitter, and Facebook. Participants in the RWCS included 44 EM residents, including three residents in Fiji. In addition, five EM attendings participated as facilitators and thre EM attendings acted as participants (Table 1). Twenty-two of 44 participants were not part of the Wellness Think Tank, and these participants provided new perspectives to inform the pre-work already done.

The five-hour event was divided between large-group presentations and small-group working time for the four consensus groups. The timeline is outlined in Figure 1. On the day of the event, updates were provided to the public via Twitter through the hashtag #RWCS.

## RESULTS

Each of the four working groups worked for seven months leading up to the one-day RWCS event to develop tangible resources that residency programs could use to improve wellness on both the group and individual level. At the summit, the small groups spent their time focusing on creating a consensus for each of the resources. The following is a brief summation of the work from each group.

### Wellness Curriculum Development

The group addressed the overwhelming request by residents in the Wellness Think Tank for a formal wellness curriculum within residency training programs. After an extensive search of the existing literature on physician wellness, resiliency, and burnout, a preliminary framework for a longitudinal and comprehensive curriculum was developed. During the summit, the working-group members reviewed the framework and recommendations. Several additional critical topics were added, including cultivating workplace wellness, dealing with difficult patients and staff, developing a support network of non-physicians, and debriefing. [WestJEM ref on RWCS Wellness Curriculum]

### Educator Toolkit for Specific Wellness Topics

Three different, comprehensive lesson plans were developed: Second Victim Syndrome, Mindfulness and Meditation, and Positive Psychology. These lesson plans focus on practical skills for residents that could be used by training programs either individually or as part of a larger wellness curriculum. During the RWCS event, the working group standardized these curricular resources to ensure that they all included active learning and that sessions lasted no more than 30 minutes. Each lesson plan concludes with a “commitment to act,” which adds an essential element of personal accountability for residents. [WestJEM ref on RWCS Educator Toolkit]

### Programmatic Innovations

Prior to the RWCS event, this small group attempted to collect a wide range of different wellness activities, resources, and ideas that had been used by residency programs across North America. During this process, it became evident that there was a diverse approach to programmatic initiatives to address each program’s unique needs. During the RWCS, in addition to cataloging the initiatives the working-group members developed a resident-level, needs assessment tool and a program-level, structured planning tool. [WestJEM ref on RWCS Innovations]

### Wellness-Targeted Technologies

A plethora of apps and other technologies focus on wellness. This group downloaded and experimented with various digital tools that tracked their steps, sleep, meditation, food habits, exercise routines, and daily mood. The focus was to identify the most relevant and practical resource for residents to maintain their wellness and resiliency. At the RWCS event, the working group collated the resources, created themes, added supplemental information for each resource (e.g. cost, platform compatibility, screenshot images), and finalized the database list.

## DISCUSSION

The RWCS event is the first step of a transformative, cultural journey focusing on resident health and wellbeing. To our knowledge, this is the first time that residents from across the world collaborated and convened to reach a consensus on these critical issues. The tools developed by the four RWCS working groups will serve as a resource for resident health and wellbeing leaders looking to influence clinical learning environments at the local organizational level for the future. Working at an organizational level is foundational but not sufficient for cultural transformation. An additional paradigm to look at resident health and wellbeing is through the paradigm of a social movement, which the Wellness Think Tank and RWCS event embody.

Veteran organizer and policy expert Marshall Ganz describes four elements necessary to lead successful social movements: relationships, story, strategy, and action.^16^ The Wellness Think Tank and RWCS have made inroads in relationships and story, and will hopefully catalyze strategy and action to ensure that resident health and wellbeing becomes a successful social movement.

### Relationships

The RWCS leadership team had experience working within the ALiEM culture prior to the RWCS. This led to the development of the Wellness Think Tank to congregate a critical mass of EM residents into a virtual community. Mirroring the ALiEM culture, the Think Tank’s culture was based on deep, reciprocal relationships that complement knowledge transactions. These relationships are facilitated by trust,^17^ communication, 18,19 and personal learning networks^20^ that allow for exponential growth. The networks developed have both strong ties that facilitate commitment and motivation, and weak ties that facilitate entry into new networks and domains.^21^ The relationships that the Wellness Think Tank and RWCS created will fuel the networks needed to implement a successful resident health and wellbeing social movement in the future.

### Story

Leading up to the RWCS event, invited EM faculty members recorded their own wellness stories. These podcast recordings were paired with blog posts discussing central wellness issues on the ALiEM website. The purpose of these stories was to normalize conversations around physician wellness and to promote an open dialogue leading up to the RWCS.

Furthermore, stories served as anchors and tangible reminders throughout the RWCS event of how resident wellness is a timely and critical issue today, especially in EM. The summit was launched with a story about a resident suicide as told by the resident’s program director. As with many stories, the RWCS was presented with a challenge (residents are burned out and committing suicide), a choice (how can the RWCS influence residents and the clinical learning environment?), and a desired outcome (increase the health and wellbeing of residents). At the RWCS conclusion, the aftermath of the resident suicide was shared with the participants.

### Strategy

The RWCS event was a unique and novel consensus meeting, involving residents as the primary stakeholders. Additionally, this summit was able to involve a greater number and wider array of participants due to the way in which the pre-work was done. A virtual community of practice, the Wellness Think Tank, was created on a digital messaging application (Slack) to allow for asynchronous collaboration for much of the summit pre-work. Using a virtual platform allowed 142 resident members across North America to engage in the process and provide feedback. In addition to the RWCS pre-work, this community also served as a means for residents to collaborate and educate themselves in various facets of wellbeing. The live event hosted 22 Think Tank members and 22 new resident participants. Involving new participants gave the working groups the opportunity for new feedback and additional input while developing their consensus projects. Next steps include disseminating the resources developed by the small groups and integrating these tools into local residency programs.

### Action

Our hope is that the residents who participated in the Wellness Think Tank and RWCS event will embrace their involvement in this grassroots movement to improve physician wellness. The group agreed that there should be a focus on developing community and open communication among physicians discussing the stressors and challenges faced by physicians in training. The RWCS has shown that it is possible to galvanize a large group of stakeholders in a relatively short time by creating robust trust, communication, and learning networks to create specific learning and tools to support resident wellness. We are hopeful that resident wellness will become a successful social movement.

## Figures and Tables

**Figure:**
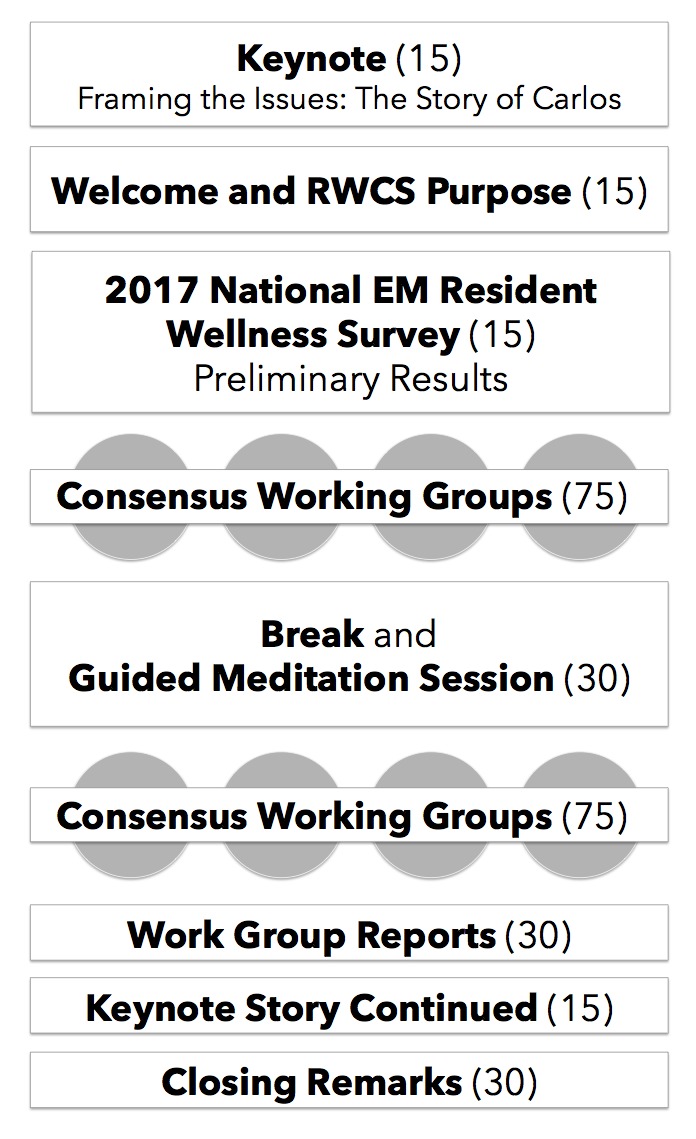
Segmented timeline of Resident Wellness Consensus Summit event and allotted time for each segment in parentheses (minutes).

**Table:** Demographic data on members of the Wellness Think Tank (WTT) and the Resident Wellness Consensus Summit.

	Wellness Think Tank	Resident Wellness Consensus Summit (WTT/non-WTT members)
Number of residents	142	44 (22/22)
Number of unique residency programs represented by residents	100	31 (30/1)
Number of attendings	12	8 (5/3)
